# Preventing the Solid Cancer Progression via Release of Anticancer-Cytokines in Co-Culture with Cold Plasma-Stimulated Macrophages

**DOI:** 10.3390/cancers11060842

**Published:** 2019-06-18

**Authors:** Nagendra Kumar Kaushik, Neha Kaushik, Manish Adhikari, Bhagirath Ghimire, Nguyen Nhat Linh, Yogendra Kumar Mishra, Su-Jae Lee, Eun Ha Choi

**Affiliations:** 1 Plasma Bioscience Research Center, Applied Plasma Medicine Center, Department of Electrical and Biological Physics, Kwangwoon University, Seoul 01897, Korea; manishadhikari85@gmail.com (M.A.); ghimirebhagi@hotmail.com (B.G.); nhatlinhusth@gmail.com (N.N.L.); 2 Laboratory of Molecular Biochemistry, Department of Life Science, Hanyang University, Seoul 04763, Korea; neha1987@hanyang.ac.kr; 3 Functional Nanomaterials, Institute for Materials Science, Kiel University, Kaiserstr. 2, D-24143 Kiel, Germany; ykm@tf.uni-kiel.de

**Keywords:** monocyte-macrophage stimulation, cancer inhibition, mesenchymal shift, cancer stem cells

## Abstract

Non-thermal atmospheric pressure plasma sources operated in ambient environments are known to generate a variety of reactive oxygen and nitrogen species which could be applied for various biomedical applications. Herein, we fabricate a micro-dielectric barrier discharge plasma device by using screen-printing technology and apply it for studying immuno-stimulatory effects. We demonstrate a tumor-suppressive role for plasma-stimulated macrophages in metastatic solid cancers that directly elicit proliferation and are responsible for tumor relapse mediated by mesenchymal shift. Using microarray analysis, we observed that cold plasma stimulates and differentiates monocyte cells into macrophages as demonstrated by expression of several cytokine/chemokine markers. Moreover, plasma treatment stimulates the differentiation of pro-inflammatory (M1) macrophages to a greater extent. These stimulated macrophages favor anti-tumorigenic immune responses against metastasis acquisition and cancer stem cell maintenance in solid cancers in vitro. Differentiation of monocytes into anticancer macrophages could improve the efficacy of plasma treatment, especially in modifying pro-tumor inflammatory microenvironment through effecting highly resistant immunosuppressive tumor cells associated with tumor relapse.

## 1. Introduction

The tumor microenvironment is well recognized to have a critical role in cancer progression and metastasis [1]. Macrophages are well-defined effector cell components of inflammation and host defense against tumors. However, tumor-associated macrophages can differentiate into either cytotoxic (M1) or tumor-promoting (M2) phenotypes depending on the tumor microenvironment [2]. Macrophages are classically activated toward the M1 phenotype by IFN-γ alone or in concert with microbial products. Alternative activation by stimulation with interleukin (IL)-4 or IL-13, IL-10, transforming growth factor-β (TGF-β), immune complexes and glucocorticoids drive macrophages toward the M2 phenotype [3]. M2 macrophages are believed to be present in most tumors and promote tumor progression [4]. Macrophages comprise the tumor-infiltrating immune cells in solid tumors [5]. For a long time, lipopolysaccharide (LPS)-activated macrophages were associated with anti-tumor approaches [6]. While the anti-cancer role of canine macrophages has been shown to rely on LPS stimulation approaches [7], similar evidence for an activated human macrophage-induced anti-cancer effect against solid cancers has not been elucidated so far. Cold atmospheric plasma (CAP) has drawn attention as a modulator in the context of biological applications and exerts a variety of functions depending upon the generated reactive species and their interaction depending on the type of cells. CAP exposure has been shown to be highly selective in anti-microbial and sterilization effects, wound healing capacity, blood coagulation and treatment of various diseases, including cancer [8,9,10,11]. However, the possible role of CAP in macrophage activation and its interaction with tumor cells remains largely obscure.

Glioblastoma multiforme (GBM) is the deadliest malignancy related to the solid tumors and central nervous system. Approximately 40% of all primary brain tumors are diagnosed in the form of a solid GBM mass with high non-homogenous infiltrative potentials [12,13], explaining the poor survival rate. Even with standard treatment technology including radiotherapy with chemotherapy for inducing tumor cell death, the patient’s median survival period is still approximately 15 months [14]. Thus, novel immune-based therapies are needed to prevent the spreading and metastasizing of solid tumor cells. Recently, we demonstrated that CAP treated macrophages release tumor necrosis factor (TNF-α), which acts as a tumor suppressor for solid tumors [15]. Hence, targeting pro-inflammatory macrophages for cancer therapy could be an attractive strategy to improve current anti-tumor treatments.

Since macrophage activation plays a crucial role in cancer by controlling the expression of genes involved in cell progression/apoptosis, we investigate the anti-tumor activity of activated human macrophages against human glioma cells and determine whether this activity can be manipulated by µ-dielectric barrier discharge (µ-DBD) plasma treatment of immune cells. Using the human, monocytic THP-1 cell line as an immune cell model, we showed that plasma-induced differentiation and polarization towards M1 pro-inflammatory macrophages limits the brain cancer invasiveness, stemness and progression. Additionally, microarray analysis confirmed that CAP-induced cytokines (M1 > M2) play a key role in phorbol 12-myristate 13-acetate-treated (PMA) activated macrophage polarization. This polarization actively suppresses tumor progression largely because of the release of TNF-α and IL-12 in co-culture conditions with tumor cells. Since, it has also been suggested that M1 macrophage could be used as a carrier for enhancing drug delivery [16], which improved therapeutic efficacy for cancer therapy, we thought to stimulate blood monocytes with macrophage differentiation using cost-effective non-thermal plasma-based stimulator. This strategy could benefit to target metastasizing cells in tumor microenvironment responsible for further cancer relapse and induced tumorigenesis. This study, for the first time, elucidates a novel plasma immunomodulation-based mechanism that promotes an anti-tumorigenic effect through modulating the monocyte-derived macrophages.

## 2. Results

### 2.1. Optimization of the Cold μ-DBD Plasma Source

**Figure 1:**
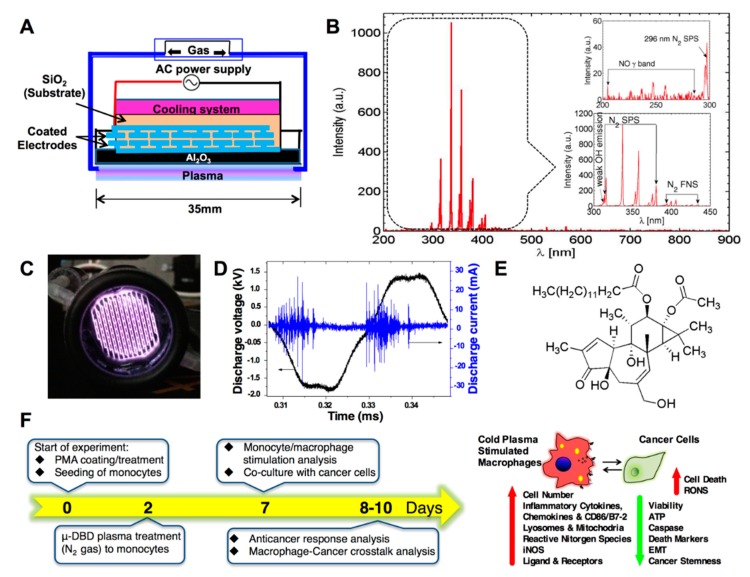
Cold μ-dielectric barrier discharge (DBD) nitrogen plasma configuration, characteristics and treatment conditions. (**A**) Schematic diagram of the μ-DBD nitrogen plasma device for biomedical applications. (**B**) Optical emission spectroscopy (OES) spectra of the μ-DBD nitrogen plasma device. (**C**) Live image of the μ-DBD nitrogen plasma device during treatment. (**D**) The voltage and current profiles of the μ-DBD nitrogen plasma device. (**E**) The structure of phorbol-12-myristate-13-acetate (PMA). (**F**) Experimental scheme for the cancer cells co-cultured with PMA-differentiated plasma stimulated macrophages. Different analysis patterns will be performed in both cancer cells and macrophages as mentioned in the right panel.

The schematic of the μ-DBD plasma source used in our experiment is shown in Figure 1A. It consists of silver electrodes (width = 200 µm, thickness = 5 µm) fabricated in a coplanar configuration above a glass substrate (SiO_2_) 35-mm in diameter and 1.8-mm thick using the technique of photolithography. The spacing between the adjacent silver electrodes was 200 µm and a 30-µm-thick SiO2 dielectric layer was coated above the electrodes. The discharge was obtained by connecting the positive and negative polarities of the power supply to either end of the coplanar silver (Ag) electrodes. Nitrogen (N2) was used as the working gas with a flow rate of 1 lpm. The temperature of the source was controlled by generating the discharge in dimming mode (on time (Ton) = 27 ms, off time (Toff) = 160 ms). The optical emission spectrum of the μ-DBD plasma source recorded with an HR4000 spectrometer is shown in Figure 1B. Emissions from the N2 s positive system (296 nm, 315.8 nm, 337 nm, 357.6 nm, 380.4 nm) and N2 first negative system (approximately 400 nm) [17], which are responsible for the formation of various reactive oxygen and nitrogen species, are clearly observed in the spectrum. In addition to this, there are weak emissions from the NO-γ band (226 nm, 236 nm, 247 nm 258 nm) and OH radical (309 nm) [17]. Figure 1C shows the photograph of the discharge captured during the Ton period. The voltage and current waveforms measured during this time are shown in Figure 1D. Plasma was generated at a frequency of 24 kHz using a 0.04-watt sinusoidal power supply. The rms values of voltage and current were 1.2 kV and 2.6 mA, respectively. In a recent report, we showed plasma induced reactions to form various kinds of reactive species in gaseous and aqueous phase [18]. We also observed that the temperature of the culture media increased to 31 °C after 5 min of plasma treatment, which is substantially below the critical physiological temperature in humans and animals. Also, our earlier studies demonstrate that this DBD plasma system is effective in reducing cell growth in various cancer types [15,19]. In this study, DBD plasma was used to stimulate PMA-adhered THP-1 monocytic cells for 1 and 3 min (Figure 1E).

### 2.2. Distinct Molecular Features Characterize a Subset of M1/M2 Cells with Plasma Treatment In Vitro

**Figure 2:**
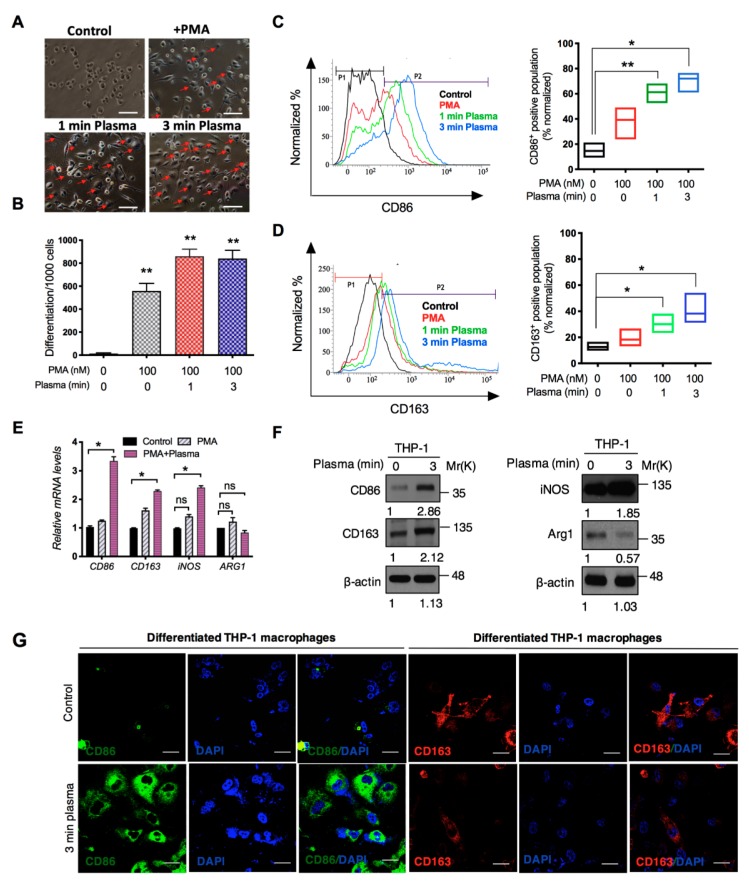

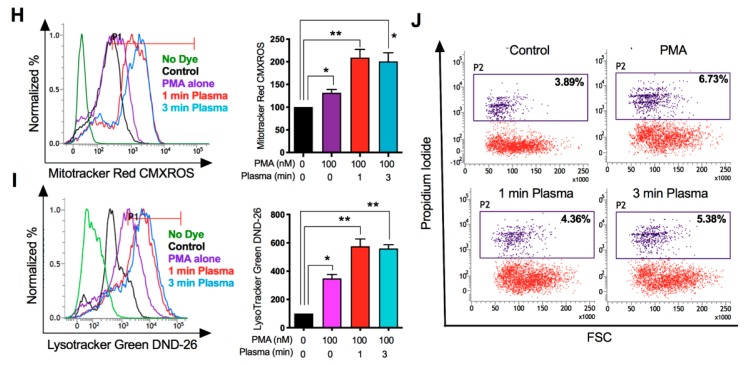
Characterization of cold plasma-stimulated macrophages. (**A**) To generate macrophage differentiation, human THP-1 monocytes were differentiated with PMA for 2 days, treated with cold DBD plasma for 1 min and 3 min on day 3 and further incubated for an additional 5 days. Polarized macrophages were observed using phase-contrast microscopy. Scale bar = 50 µm. (**B**) The quantitative determination of plasma-differentiated THP-1 cells is shown in the bar graph. (**C**,**D**) After plasma treatment, cell surface expression of CD86 (M1 marker) and CD163 (M2 marker) was analyzed by flow cytometry as shown in the histograms. Representative graph indicates the changes in macrophage cell surface markers followed by DBD plasma treatment. (**E**) QPCR analysis of the mRNA expression of the CD86, CD163, iNOS and ARG1 genes after 3-min plasma treatment in PMA-stimulated THP-1 cells. (**F**) Western blot analysis of the protein expression of the CD86, CD163, iNOS and ARG1 genes after control and 3-min plasma treatment in PMA-stimulated THP-1 cells. β-actin was used as a loading control. (**G**) Immunofluorescence staining of CD86 and CD163 in plasma-stimulated THP-1 cells. Scale bar = 10 µm. (**H**,**I**) The numbers of mitochondria and lysosomes were analyzed by staining with MitoTracker Red CMXROS and LysoTracker Green DND-26, followed by FACS analysis. (**J**) Cell death was assessed by propidium iodide (PI; 50 ng/mL) in PMA-treated THP1 cells after 1 min and 3 min of plasma treatment. Error bars represent mean ± S.D. of triplicate samples. * *p* < 0.05 and ** *p* < 0.01. Uncropped images are shown to Figure S1.

The potential of non-thermal cold plasma to cause macrophage activation in monocytes model population was investigated. THP-1 monocytic model cells were differentiated with PMA treatment prior to plasma treatment and exposed to 1 and 3 min plasma after 48 h of PMA treatment. Following plasma treatment, these cells were allowed to differentiate further for 5 days to obtain differentiated cells (Figure 1F). As we mentioned earlier, PMA was completely removed by adding fresh medium to culture before plasma exposure to check direct plasma effect on macrophage activation. As shown in Figure 2A,B, compared with the differentiation of native cells and PMA-treated cells, 1 and 3 min treatment doses of plasma exposure increased the differentiation of monocytic cells. Previous reports showed that M1 macrophages express excessive levels of inducible nitric oxide synthase (iNOS), CD68, CD80 and CD86 costimulatory molecules [20,21]. In contrast, M2 macrophages have been recognized to express CD163 (scavenger receptor) and CD206 (mannose receptor) as anti-inflammatory markers and Arginase-1 (ARG-1) [20,22]. In line with these studies, we next questioned whether plasma can polarize macrophages towards M1 and M2 type. To this end, we performed flow cytometry analysis to determine the M1- and M2 positive cell population. Flow cytometry analysis showed that the number of CD86 (M1 macrophage marker)-positive cells was significantly higher in PMA treated plasma-treated macrophages than the CD163 (M2 macrophage marker) population when compared with only PMA treated groups (Figure 2C,D). At the same time, CD86 and iNOS mRNA levels were highly elevated compared to control in plasma-treated THP-1 cells; however, the ARG1 levels were not significantly different between the only PMA- and PMA-plasma-treated groups (Figure 2E). Consistent with the above findings, western blot data confirmed that the CD86 and iNOS protein levels were increased in the 3-min plasma-treated THP-1 cells (Figure 2F). In addition, other markers CD80, CD68 and CD206 markers were also elevated in plasma stimulated THP-1 cells at 3 min exposure (Figure S2). The TCGA database further validated that the NOS levels are also higher in brain carcinoma tissues including mesenchymal, neural and proneural subtypes (Figure S3).

In addition, we also observed higher CD86 receptor (M1 marker) expression in plasma-exposed THP-1 cells than in the native cells. In contrast, low CD163 receptor expression levels were detected in THP-1 cells after plasma treatment as confirmed by immunofluorescence (Figure 2G). From these data, we conclude that plasma exposure increased M1-positive population in PMA treated THP-1 cells than only PMA-treated cells. Another feature of macrophage differentiation is increased number of certain membrane-bound organelles [23,24]. To confirm the increased numbers of cellular organelles in the cytoplasm, we next stained plasma treated PMA-activated THP-1 cells for mitochondria and lysosomes, whose cytoplasmic number contributes to the differentiation and accumulation of macrophages after the stimulus [25]. Flow cytometer analysis revealed that the plasma-treated THP-1 cells had greater intensity of mitochondrial and lysosomal staining than the native groups (Figure 2H,I). These observations clearly suggested that plasma exposure effectively induced macrophage polarization/differentiation in THP-1 cells.

### 2.3. M1-Like Macrophages Induce Solid Tumor Cell Death If Activated by Plasma with PMA

**Figure 3:**
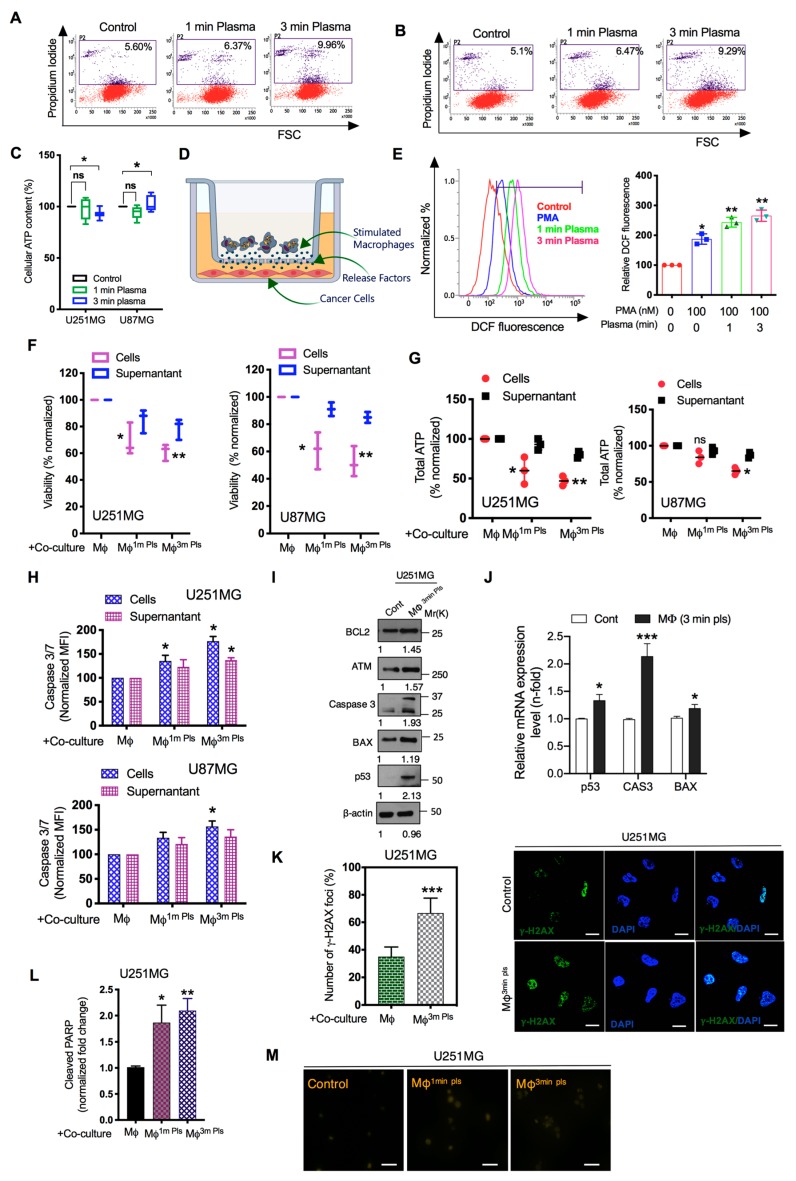
Macrophage-cancer cell co-culture induced glioma cell death. (**A**,**B**) U251MG and U87MG brain cancer cell death was determined by PI (50 ng/mL) staining after only 1 min or 3 min of plasma treatment. (**C**) Cellular ATP content was measured using a commercially available kit in only plasma-exposed U251MG and U87MG brain cancer cells. (**D**) The schematic diagram for the tumor-macrophage cell co-culture. Plasma-stimulated THP-1 macrophages (1 × 10^5^ cells/mL) were seeded in transwells (0.4-µm porous membrane), while cancer cells were seeded in 6-well plates at a concentration of 2 × 10^5^/insert 24 h prior to the co-culture experiment. (**E**) DCF fluorescence was observed in glioma cells after co-culture with stimulated macrophages as per indicated panels using flow cytometry. Reactive oxygen nitrogen species were detected using H2DCFDA (10 µM) in glioma cells after 24 h incubation. Quantification of the FACS values is shown in the graph. (**F**) In the co-culture condition with 1- and 3-min plasma-stimulated macrophages, the U251MG and U87MG cell viability were determined using colorimetric MTT assays. (**G**) Cellular ATP was also determined in both cancer cells in a similar co-culture condition. (**H**) The representative graph indicates the cleaved caspase-3/7 activity in both glioma cell lines co-cultured with 1-min and 3-min plasma-stimulated macrophages. (**I**) Western blot analysis of pro-apoptotic markers in U251MG cells when co-cultured with 3-min plasma-stimulated macrophages. (**J**) q-PCR analysis for p53, CAS3 and BAX genes in U251MG cells when co-cultured with 3-min plasma-stimulated macrophages. (**K**) γ-H2AX, a marker of DNA double-stranded breaks, was visualized in U251MG cells after co-culture with 3-min plasma-stimulated macrophages. Quantification of the number of observed γ-H2AX foci is shown in the graph. Scale bar = 5 µm. (**L**) Cleaved PARP in U251MG cells was assessed by ELISA assay after co-culture. (**M**) Apoptosis was analyzed in co-culture-treated glioma cells by TUNEL assays. These analyses were performed after 48 h incubation in the post-co-culture setup. The control group represents cells only treated with PMA for macrophage differentiation without plasma exposure. β-actin was used as a loading control. Scale bar = 100 µm. Error bars represent the mean ± S.D. of triplicate samples. * *p* < 0.05 and ** *p* < 0.01. Uncropped images are shown to Figure S4.

Next, we investigated the possible contribution of plasma-activated macrophages towards anti-cancer activity. Prior to these experiments, we confirmed that plasma treatment did not induce significant cell death in monocytic cells using propidium iodide (PI) staining (Figure 2J). On the other hand, MTT assays showed that a single plasma exposure had the least effect on cell death in U251MG and U87MG solid cancer cells (Figure 3A,B). Given that ATP is the central energy source of cells and, therefore, a measure of cellular metabolism and viability, we have further investigated the cellular ATP content in glioma cells using cell-titer Glo reagent. The ATP levels of glioma cells were differentially affected by plasma treatment alone in both types of glioma cells, as seen in Figure 3C. Thus, the differential affected ATP levels could be explained by a change in viability induced by plasma treatment in PMA treated THP-1 cells. To observe direct evidence of plasma-stimulated macrophages, we co-cultured these plasma stimulated macrophages with GBM cells, as depicted in Figure 3D. As plasma has been widely shown to induce cell death through ROS [18,26]. We first detected intracellular ROS levels in glioma cells in co-culture condition with plasma stimulated macrophages. The plasma-treated groups had higher levels of ROS in glioma cells than the control groups (Figure 3E). Only PMA-treated THP-1 cells were also able to induce ROS in glioma cells in co-culture conditions; however, this effect was boosted by plasma treatment at 1- and 3-min exposure. After two days, the number of viable tumor cells was measured by MTT assays in the same co-culture condition. Plasma-activated macrophages directly affected the cell viability and ATP content of U251MG and U87MG cells compared with those observed in the co-culture condition with supernatant medium (Figure 3F,G). Moreover, caspase-3/7 activation (an indicator for apoptotic cell death) was also increased by the direct co-culture condition in glioma cells (Figure 3H). The growth inhibitory effect of the activated macrophages on glioma cells was also examined by screening the anti-apoptotic gene levels. There was a significant induction of BCL-Xs, ATM, BAX, cleaved caspase-3 and p53 expression in U251MG cells when co-cultured with plasma-stimulated macrophages as seen by western blotting (Figure 3I). Consistently, mRNA levels of p53, CAS3 and BAX were also upregulated in glioma cells when co-cultured with plasma stimulated macrophages in similar conditions (Figure 3J). In addition, the histone 2A family member X (γ-H2AX) is known to be phosphorylated at serine 139 and forms discrete foci at the DSB sites in response to DNA double-stranded breaks (DSBs) during apoptotic cell death [27]. Accordingly, we next stained glioma cells with γ-H2AX dye after co-culture with the macrophages and found that the amount of DSBs was highly increased in glioma cells (Figure 3K). Notably, cleaved Poly(ADP-ribose) polymerases (PARP1) activity, which is shown to be activated by DNA damage [28] was also elevated in glioma cells in the co-culture condition as confirmed by ELISA assay (Figure 3L). TUNEL analysis (apoptotic cells marker) further validate our observations that apoptotic cells were significantly higher in co-cultured glioma cells (Figure 3M). Our data demonstrate that differentiation of human macrophages into pro-inflammatory M1 with plasma leads to apoptotic cell death through inducing genes involved in DNA damage checkpoint and cell cycle arrest which could be capable of inhibiting glioma cell growth for anti-tumor approaches.

### 2.4. Analysis of the Cytokine Profile Expressed by Macrophages

**Figure 4:**
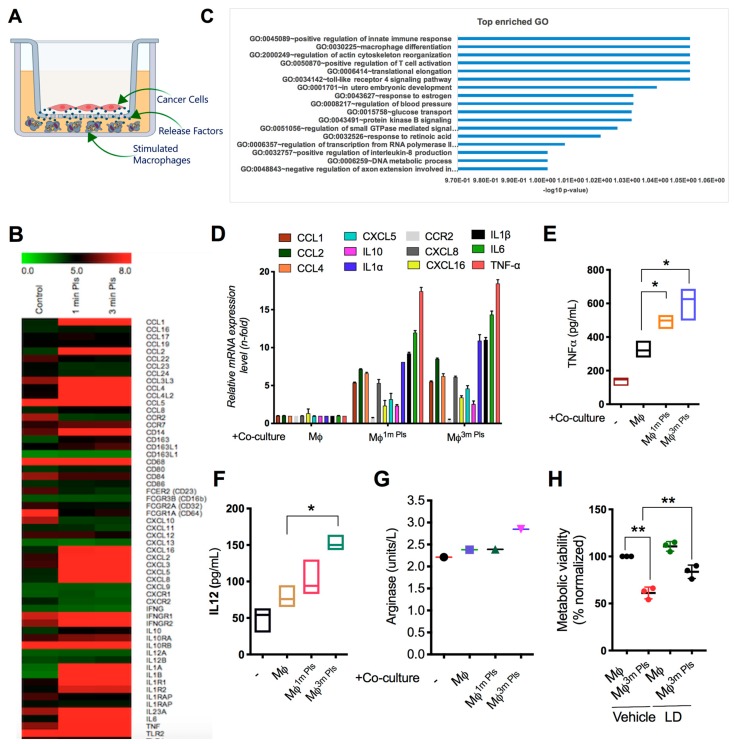

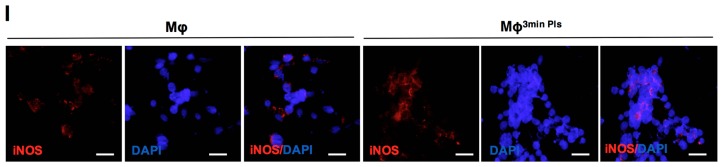
Expression of cold plasma-stimulated macrophage surface markers and release of cytokines by macrophages exposed to the plasma through M1 and M2 polarization. (**A**) Schematic diagram for the detection of released secretion factors during tumor-macrophage cell co-culture. To this end, plasma-stimulated THP-1 macrophages (1 × 10^5^ cells/mL) were seeded in the lower chamber of the same transwell as mentioned above, while cancer cells were seeded on the top of the insert. (**B**) Microarray analysis was performed on plasma-stimulated macrophages and expression data were generated by Affymetrix Expression Console software version 1.1. To classify the co-expression gene group with a similar expression pattern, we performed hierarchical clustering using MEV (MultiExperiment Viewer) software 4.4 (www.tm4.org). More than 2-fold up genes are shown in Figure 4B (**C**) Go analysis of various signaling molecular pathways involved in altered gene expression of plasma-stimulated macrophages. (**D**) qPCR was used to validate the gene changes (related to M1 and M2 polarization) in plasma-stimulated macrophages. (**E**,**F**) The release of cytokines (TNF-α and IL-12) was evaluated in cell culture media after 24 h of co-culture using an ELISA assay in THP-1 treated cells as per the indicated panels. (**G**) Arginase-1 (ARG-1) was analyzed in cell culture media after 24 hrs. of co-culture using an ELISA assay in THP-1 treated cells as per the indicated panels. (**H**) Cellular metabolic viability was determined in U251 MG cells when co-cultured with plasma-stimulated macrophages in the presence of lenalidomide (2 µM, LD), a TNF-α inhibitor, after 48 h. (**I**) Immunocytochemistry of control and 3-min plasma-treated macrophages for iNOS detection. Control group represents cells only treated with PMA for macrophage differentiation without plasma exposure. Scale bar = 100 µm. Error bars represent the mean ± S.D. of triplicate samples. * *p* < 0.05 and ** *p* < 0.01.

Given that receptors expressed, and cytokines secreted, by macrophages could provide a clue about their polarization state, we next sought to investigate the mechanisms involved in the anti-cancer activity of plasma-stimulated macrophages. To this end, we placed plasma-treated macrophages in the lower chamber co-cultured with glioma cells as illustrated in Figure 4A and performed microarray analysis. The transcriptome profile was evaluated in the control and plasma-treated THP-1 cells. A Venn diagram shows the number of overlapped genes upregulated and downregulated by plasma treatment (Figure S5A) in plasma-activated macrophages. Additionally, PCA analysis was applied to the whole dataset and it was validated that 100% of the total variance of the system lies within the first two components. This analysis revealed that macrophage polarization was associated with a modification of the global transcriptome profiles (Figure S5B). Using a heat map analysis, we showed that plasma-treated macrophages differentially expressed various genes involved in M1 and M2 polarization (Figure 4B). Interestingly, GO analysis revealed that pathways involved in macrophage differentiation and immune response were highly activated by plasma treatment in THP-1 monocytes (Figure 4C). Consistent with microarray data, real-time PCR data confirmed that co-culture of plasma-treated macrophages with glioma cells increased various cytokines associated with M1 and M2 markers (Figure 4D). Among all, plasma exposure remarkably increased the expression of IL-1α, IL-1β, IL-6 and TNF-α. It was worth mentioning here that TNF-α (M1 marker) was highly increased in 3-min plasma-exposed macrophages in the co-culture condition to a greater extent (Figure 4E). These data indicate that plasma treatment may stimulate genes associated with M1-type pro-inflammatory macrophages. Activation of macrophages enhanced the production of TNF-α and IL-12 after two days of co-culture with tumor cells (Figure 4F,G) as detected by ELISA assay, while ARG1 remained unchanged among all panels except for the 3 min plasma-exposed macrophages (Figure 4H). We also found that plasma-treatment increased IL-12 in cell-culture medium in the co-culture condition (Figure S6A). The data above showed that the expression of TNF-α is uniquely modified by plasma treatment involved in the induction of pro-inflammatory activation pathway. To determine the possible role of TNF-α in controlling cell viability, we investigated U251MG cell viability in the presence of lenalidomide, a TNF-α inhibitor. The TNF-α inhibitor significantly blocked the reduction in cell viability induced by stimulated macrophages. Because iNOS levels increase during M1 macrophage differentiation, we stained for iNOS in plasma-stimulated macrophages. We observed that iNOS expression was higher in plasma-stimulated macrophages than in controls after co-culture with glioma (Figure 4I). A similar pattern was observed when analyses with qPCR under same conditions (Figure S6B). Collectively, these data suggested that the inhibition of cancer cell growth might be mediated through the expression of those genes induced by pro-inflammatory M1 macrophages.

### 2.5. Co-Culture of Cancer Cells with Plasma-Activated Macrophages Decreases the Invasive Behavior of Cancer Cells

**Figure 5:**
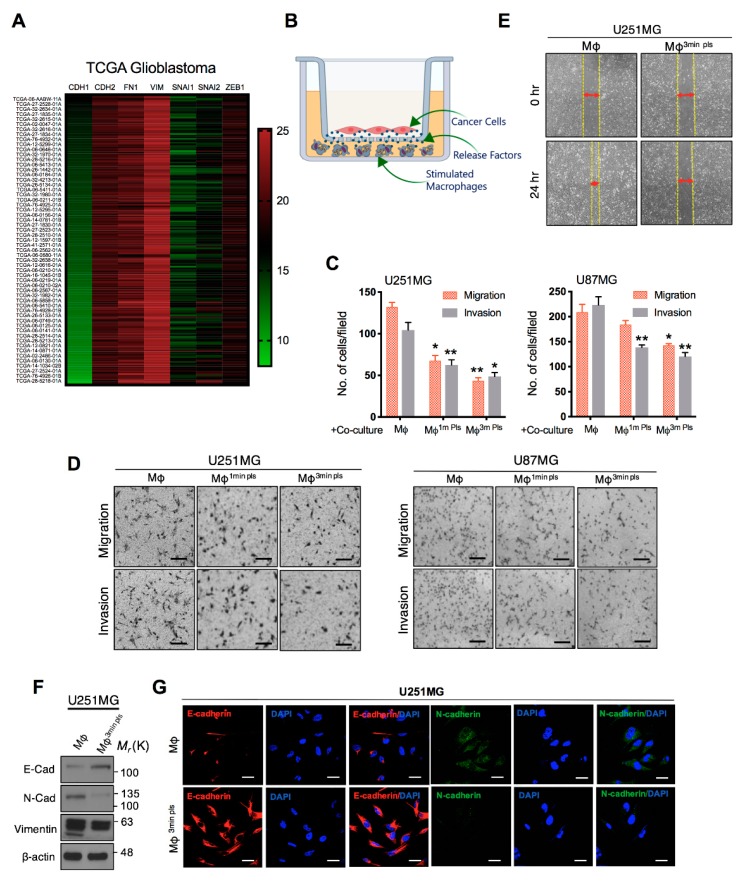
Analysis of the epithelial-mesenchymal transition in glioma cells after macrophage-cancer cell co-culture. (**A**) UCSC Genome Browser showing the expression of EMT signature genes in glioblastoma extracted from TCGA database. (**B**) Schematic diagram for tumor-macrophage cell co-culture and conditioned as mentioned in Figure 3. (**C**,**D**) Bar graph and representative images of the Transwell assays for migration and invasion in U251MG and U87MG brain cancer cells 48 h after co-culture treatment, Scale bar = 100 µm. The bar graph shows the number of cells selected from three independent fields of each group. (**E**) Wound-healing (Scratch assay) assay to analyze the migration of U251MG cells after co-culture in a similar condition. (**F**) Immunoblotting for EMT-indicative markers in glioma cells after co-culture with control and 3-min plasma-stimulated macrophages. (**G**) Confocal images of E-cadherin and N-cadherin expression in glioma cells after co-culture, scale bar = 10 µm. Error bars represent the mean ± S.D. of triplicate samples. * *p* < 0.05 and ** *p* < 0.01.

It has been believed that during later stages of carcinogenesis, epithelial-mesenchymal transition (EMT) also contributes to many malignant features of cancer cells, including anti-apoptotic, motile, invasive and stem-like features [29]. To this end, we have extracted the data from patient samples through the University of California Santa Cruz (https://xena.ucsc.edu) and TCGA analysis showed that mesenchymal signature genes are highly upregulated in high-grade gliomas (Figure 5A). In line with these studies, following the observation of apoptosis above in glioma cells by plasma-activated macrophages in co-culture, we questioned whether plasma-activated macrophages could induce changes at the level of EMT abundance in co-cultured glioma cells. To this end, glioma cells were co-cultured in the upper chamber of a transwell in the presence of plasma-polarized macrophages as shown in Figure 5B. After 48 h of co-culture, glioma cells were removed and tested for migration and invasion phenomenon. The transwell Boyden chamber and wound-healing/scratch assays showed that 3-min plasma-stimulated macrophages effectively suppressed the migration and invasion of glioma cells (Figure 5C–E). EMT is characterized by the loss of epithelial cell junction proteins and the gain of mesenchymal markers. Accordingly, we observed protein expression of epithelial marker E-cadherin was upregulated in U251MG cells in the co-culture condition. Also, the protein expression of N-cadherin and Vimentin, mesenchymal markers, were downregulated in U251MG cells after co-culture with plasma-stimulated macrophages (Figure 5F). Similarly, a gain in E-cadherin and loss of N-cadherin marker expression were confirmed by immunofluorescence (Figure 5G). Thus, the co-culture of plasma-stimulated macrophages increased the expression of epithelial markers to prevent EMT events in GBM cells. These results may highlight a crucial role of plasma stimulated macrophages in response to EMT.

### 2.6. Plasma-Polarized Macrophages in the Co-Culture Condition Effects the Stemness of Cancer Stem-Like Cells

**Figure 6:**
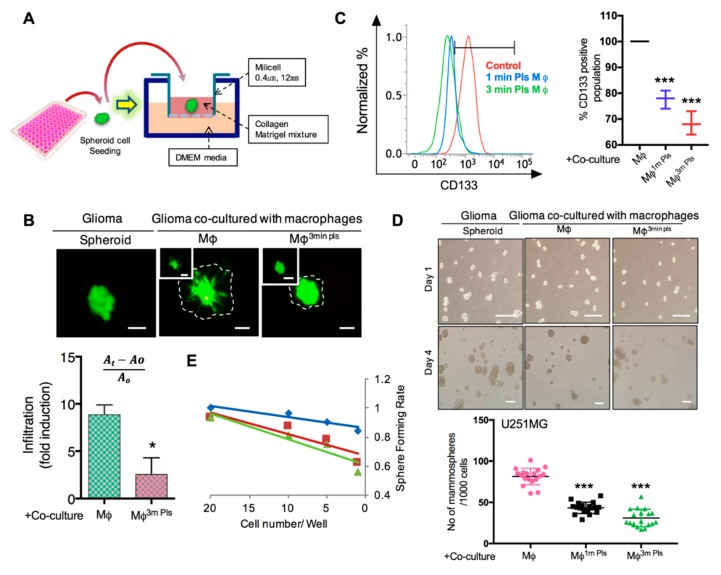
Co-culture effect on cancer-stem-cell maintenance. (**A**) Diagram displaying the set up for the 3D culture to measure glioma sphere cell invasiveness. (**B**) Images showing gliospheres after being co-cultured with plasma-stimulated macrophages in a collagen-matrigel-based matrix system. A0 denotes the area of a sphere at day 0 while At is the area on day 4. (**C**) Flow cytometry for the CD133-positive population in glioma spheroids co-cultured with control and 3-min plasma-stimulated macrophages. (**D**) Sphere-formation assay in U251 spheres co-cultured with control and plasma-stimulated macrophages. (**E**) A limiting dilution assay (LDA) was performed on U251 sphere cells in similar co-culture conditions. Solid lines represent the average value of the samples. Scale bar = 50 µm. Error bars represent the mean ± S.D. of triplicate samples. * *p* < 0.05, ** *p* < 0.01 and *** *p* < 0.001.

Brain cancer-stem-like cells are believed to exist in glioma tissues [30] and these sub-populations of cancer-like stem cells show resistance to available current therapies [31]. Therefore, we next sought to test the effect of plasma-stimulated macrophages on sphere-cultured glioma cells. To determine the effect of plasma-stimulated macrophages on the invasiveness of cancer stem-like cells in glioma, we designed a 3D culture system in which collagen type I, a common extracellular-matrix component and matrigel, with a composition like that of the basement membrane, were mixed together and solidified in their respective cell growth medium (Figure 6A). Prior to the experiment, U373s GBM cells were transduced with green fluorescence protein for their visualization. After 48 h of co-culture with plasma stimulated macrophages, GBM spheroids were plated in 3D culture system as mentioned before to their check invasive potential. Interestingly, these macrophages decreased the invasion of GBM spheroids after 3 min of plasma treatment in 3D culture (Figure 6B). Since the glioma stem-like subpopulation has a high expression of CD133 and displays a higher carcinogenic ability [30], we measured the CD133-positive population in GBM cells after co-culture with 3-min plasma-stimulated macrophages. Flow cytometer analysis showed that the number of CD133-positive cells was lower in the co-culture condition in U373 sphere cells than in the control condition (Figure 6C). To confirm the ability of plasma-stimulated macrophages to downregulate the cancer stem-like cell population in glioma cells, we subsequently performed sphere-forming and clone-forming assays in U373 glioma sphere cells after co-culture with plasma-stimulated macrophages. To this end, following a 48-hr co-culture, sphere cells were seeded for testing sphere formation and clone formation. Remarkably, we observed that co-culture with stimulated macrophages attenuated the self-renewal of the glioma stem-like population as confirmed by the sphere-formation and limiting-dilution assays (Figure 6D,E and Figure S7). These results clearly demonstrated that plasma-polarized macrophages have the potential to inhibit cancer stem cell-like traits in solid tumor cells.

### 2.7. Plasma Attenuates Mesenchymal Markers In Vivo

**Figure 7:**
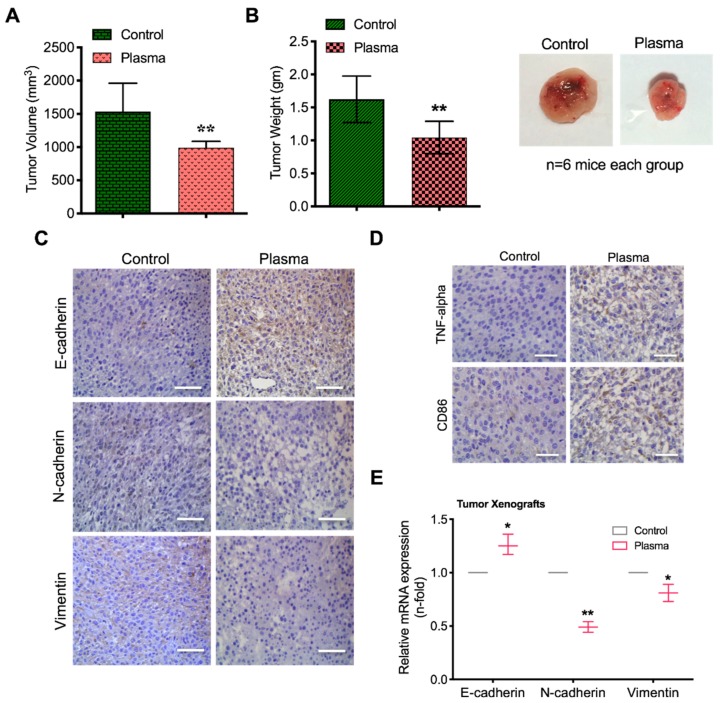

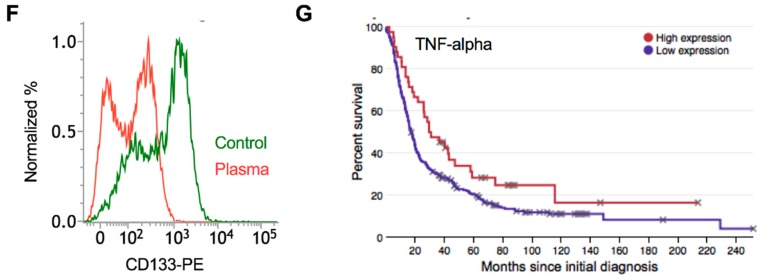
Plasma treatment shows efficacy to suppress tumorigenic potential in vivo through macrophage stimulation. (**A**) U87 glioma cells were subcutaneously injected in mice and tumor volume was calculated in control and plasma-treated groups. Plasma-treated medium (PAM) was prepared with 10 min plasma treatment. (**B**) Tumor weight was also estimated in similar groups as mentioned above. Represented images were displayed in the right side. (**C**) IHC images of EMT markers such as E-cadherin, N-cadherin and Vimentin in control and plasma treated groups. (**D**) DAB staining images of TNF-α, CD86 (M1 macrophage marker) in control and plasma treated groups. (**E**) qPCR analysis of E-cadherin, N-cadherin and Vimentin gene expression. (**F**) Flow cytometry for the CD133-positive population in glioma cells extracted from mice xenografts. (**G**) TCGA analysis of glioma patient’s cohorts based on low and high expression of TNF-α. Scale bar = 50 µm. Error bars represent the mean ± S.D. of triplicate samples. * *p* < 0.05 and ** *p* < 0.01.

Finally, to investigate the inhibitory effects of plasma exposure on tumorigenesis in vivo, the U87 glioma cells were subcutaneously injected into the flanks of balb/c female nude mice (*n* = 6 each group). PAM was prepared for mice treatments as mentioned previously in material method section. After 9 days, PAM was injected intratumorally four times every next days into the glioma xenograft mice models. Our data showed that the tumor volume in plasma treated mice was dramatically reduced compared to the vehicle control treated group (Figure 7A). Notably, after sacrifice, there was a remarkable difference in tumor weight also which was comparable to untreated control groups (Figure 7B and Figure S8). Consistent with our in vitro studies, interestingly, treatment of plasma increased the TNF-α and CD86 expression (M1 macrophage recruitment marker) in treated mice with a subsequent decrease in N-cadherin (mesenchymal marker), CD133 (CSC population marker) (Figure 7C–F). Moreover, we have extracted the patient prognosis survival data from TCGA survival database and confirmed that high expression of TNF-α improves glioma patient’s survival (Figure 7G). Collectively, our findings indicate that plasma treatment has the potential to suppress tumorigenesis by stimulation of macrophages in vitro as well as in vivo.

## 3. Discussion

The present study was designed to investigate the effect of cold plasma on macrophage activation, as macrophages are essential modulators of the tumor microenvironment and could be recruited after radiotherapy [32]. Here, we characterized the response of PMA-treated differentiated monocyte cells to cold plasma exposure at low doses. In this study, we used PMA-stimulated macrophages differentiated from THP-1 monocytes as a model because they are considered to model macrophage function since primary tissue macrophages cannot be freely expanded ex vivo [33]. We show that cold plasma-stimulated macrophages can interfere with cancer cell progression. Inhibition of brain cancer cell growth by activated macrophages was mediated by various soluble factors and cytokines induced upon macrophage activation after plasma treatment. Additionally, we report that plasma-stimulated macrophages could exert anti-cancer activity by controlling the cancer stem-like population in glioblastoma cells.

Activated/polarized macrophages were categorized by their differential expression of CD surface markers. M1-type macrophages expressed high levels of the CD86 marker, a co-stimulatory receptor necessary for T cell activation, while M2-type macrophages exhibited higher expression of scavenger receptor CD163, involved in the endocytosis of hemoglobin [34]. Here, we found that cold plasma exposure could stimulate the expression of both CD86 and CD163 markers in THP-1 cells; however, CD86 expression was significantly higher than CD163 expression (Figure 2). The plasma-stimulated macrophages were further characterized by increased numbers of mitochondria and lysosomes in plasma-exposed THP-1 cells, which contribute to the differentiation and accumulation of macrophages after a stimulus [22,23]. The differential expression of various cytokine and surface receptor panels produced by M1 and M2 macrophages revealed their functions as either inhibitory or stimulatory modulators of immune responses and inflammation. M1 is believed to represent a pro-inflammatory phenotype with consequent production of pro-inflammatory cytokines such as TNF-α and IL-12, as well as IL-1β and stimulation of inflammation [35]. In general, cytokines released by M1 macrophages serve as moderators in many inflammatory and autoimmune diseases. We have also recently shown that macrophage-secreted TNF-α acts as a critical player for cancer cell death after cold plasma treatment [15]. Meanwhile, TNF-α has been suggested to confer anti-cancer properties [36] and was also produced by plasma-polarized macrophages during the co-culture (Figure 4D). It is worth mentioning here that plasma-treated macrophage-secreted soluble factor showed the efficient release in co-culture to inhibit cancer cell growth as visualized by ELISA assay. In line with these studies, blocking TNF-α activity using the chemical inhibitor lenalidomide conquered the activated macrophage-induced cell death in glioma cells (Figure 4H). Altogether, these data suggest a role for TNF-α as a critical factor for macrophage-induced glioma cell death after plasma treatment. Galvan-Pena group suggested that NOS2 expressed by M1 macrophages plays a key role in antimicrobial activity and can function as an effective pro-inflammatory facilitator in mouse macrophage [37]. In this study, we observed that iNOS was upregulated in plasma-activated macrophages. Apart from this, M2 macrophages are significant for the progression of various diseases. These macrophages produced relatively low levels of TNF-α and IL-12 and high amounts of IL-10, imitating their anti-inflammatory phenotype and likely acting to support tissue repair mechanisms. Expression of IL-10 regulates the function of M2 macrophage. Likewise, IL-10 has been reported to be involved in the inhibition of pro-inflammatory cytokine synthesis, NO release and the control of ARG-1 expression, which regulates cell growth [38]. We also observed that IL-10 and ARG-1 expression was slightly increased due to plasma exposure in differentiated macrophages. These findings indicate that the plasma-dependent polarization of both M1 and M2 may indeed affect the pro-apoptotic function of macrophages; however, this effect could be due to a high M1/M2 ratio.

The transition from epithelial to mesenchymal markers has been a widely accepted phenomenon in carcinogenesis [39]. Frequent tumor relapse is now a major hurdle in treating aggressive forms of cancers. In this study, we revealed that plasma-stimulated macrophages efficiently decreased the migratory and invasive traits of glioma cells after co-culture (Figure 5). This approach was supported by cancer stem cell studies in which glioma stem-like cells were co-cultured with plasma-stimulated macrophages and tested for self-renewal properties. These stem cell populations usually reside within tumor cells and are the major cause of tumor recurrence after existing therapies [30]. The significance of the plasma-stimulated macrophages was identified in our studies using sphere-forming and clone-forming assays, which showed that plasma-differentiated macrophages reduced these populations along with a reduction in CD133-positive cells and disturbed glioma stem cell maintenance both in vitro and in vivo ( Figure 6 and Figure 7). In vivo data studies also demonstrated that EMT phenomenon was abrogated in xenografts mice models (Figure 7). Collectively, the efficacy of activated macrophages in cancer cell death after plasma treatment could be validated by these observations. These plasma-stimulated macrophages play a critical role in the destruction of glioma cells, glioma-like stem cell maintenance and EMT progression. Plasma-activated macrophage polarization could be a promising approach for the treatment of aggressive brain cancer diseases depicted by a compromised immune response state.

In conclusion, we demonstrate that cold plasma can function as an immune modulator for immune cell activation that can stimulate M1/M2 macrophage polarization. Particularly, the numbers of mitochondria and lysosomes were increased during monocyte cell differentiation after plasma stimulation and cell surface marker CD86 expression was elevated. Simultaneously, the induction of both macrophage subsets demonstrated the ability of plasma to induce polarization of macrophages. However, TNF-α and IL-12 were found to increase cell death by activated macrophages, which might be the results in the reduction of cellular ATP in GBM cells. Particularly, these M1-polarized macrophages may delay the EMT process and increase expression of the epithelial marker E-cadherin in glioma cells in vitro as well as in vivo. Plasma-polarized macrophages attenuated cancer stem cell maintenance, suggesting a potential role of plasma in switching macrophage function. Delays in the function of anti-inflammatory M2 and increased function of pro-inflammatory M1 macrophages might be key targets in controlling tumorigenesis caused by compromised immune responses.

## 4. Materials and Methods

### 4.1. Human Cell Culture and Differentiation

THP-1, U251MG and U87MG cells were obtained from Korean Cell Line Bank (Seoul, Korea) and cultured as per the supplier’s instructions. THP-1 monocytes differentiation was initiated by adding 100 ng/mL PMA (Sigma-Aldrich, Seoul, Korea) in RPMI-1640 medium [15]. For gliosphere cultures, cells were cultured in serum-free DMEM-F12 medium as described in our previous report [19]. After the addition of PMA, cells were allowed to differentiate for 2 days followed by plasma treatment. A control sample of differentiated monocytes without plasma treatment was included as a negative control in each set of experiments.

### 4.2. Macrophage-Tumor Cell Co-Cultures

After seven days of differentiation (2 days incubation with PMA and 5 days incubation after plasma exposure), the supernatants of the THP-1 macrophage cell cultures were collected. Adherent macrophages were washed and detached by accutase (Sigma-Aldrich) treatment for approximately 20–25 min at 37 °C. Glioma cells were seeded in 6-well plates in DMEM medium at 20,000 cells (cancer cell survival/migration assay) per well and incubated for cell attachment. After 4 h, 20,000 differentiated monocyte cells were added in the transwell (0.4 µm pore size) placed in the same 6-well cell culture plate and the macrophage-tumor cell co-cultures were incubated for the next 24–48 h. In the cytokine detection experiments, co-cultures were performed in the presence of tumor cells in the transwell while differentiated macrophages were adherent in the 6-well flat-bottom plate surface prior to the co-culture.

### 4.3. Flow Cytometry

Stimulated THP-1 Cells were prepared and incubated with the CD163-PE antibody (ab95613), CD86-PE antibody (ab77226), Mitotracker red CMXRos (M7512, Molecular probes) and Lysotracker Green DND-26 (L7526, Molecular probes) according to the manufacturer’s instructions. Stained cells were profiled using a BD FACSVerse cytometer and the FACS suite software. For the analysis of cell death, the cell suspensions were stained with propidium iodide (PI) and stained cells were analyzed by flow cytometry. To measure CD133 expression in glioma cells, the tumor cells were disassociated with accutase treatment and then the cells were labeled with an anti-CD133/1-PE antibody (AC133 clone; Miltenyi Biotec, San Diego, CA, USA) and analyzed. For the detection of reactive oxygen species (ROS), the cells were washed and incubated with 10 μM of H2DCFH-DA (Molecular probes, Invitrogen, Waltham, MA, USA) for 40 min at 37 °C in dark and analyzed.

### 4.4. Cytokine ELISA

Culture supernatants from differentiated macrophages were harvested after the required time and the concentration of arginase 1, TNF-α and IL-12 was assessed by ELISA using the commercially available assay kits (Biovision Inc., San Francisco, CA, USA) according to the kit manufacturer’s instructions.

### 4.5. Caspase-3 and ATP Assays

After co-culture with macrophages, intracellular ATP levels were measured using an EnzyLight™ ATP assay kit (Bioassay Systems, Hayward, CA, USA) in glioma cells. All treated samples were normalized to the untreated control cells. Caspase-3/7 activity was detected using a Caspase-Glo 9 assay kit (Promega, Madison, WI, USA) according to the manufacturer’s protocol.

### 4.6. Confocal Microscopy

Briefly, cells were fixed with 4% PFA and permeabilized. Immunofluorescence staining was performed at an antibody dilution of 1:200 in blocking buffer containing NP-40 and FBS in PBS. CD86, CD163, γ-H2AX, iNOS, E-cadherin and N-cadherin was stained with Alexa Fluor^®^ 488 and nuclei were counterstained with DAPI mounting media (ProLong Gold antifade reagent, Thermofisher Scientific, Seoul, Korea). The stained images were observed at 60× magnification using a fluorescence microscope (Ti–U, Nikon, Tokyo, Japan).

### 4.7. Migration and Invasion Assays

Briefly, we performed invasion assays in Transwell Boyden chambers with inserts that were precoated with growth factor-reduced Matrigel (BD Biosciences, Franklin, NJ, USA). Here, 2 × 10^4^ cells per well were placed in the upper chamber of a Transwell chamber in serum-free DMEM. The cells were then allowed to migrate toward the lower chamber, in which DMEM containing 10% FBS was placed, for 20–24 h at 37 °C. Migrated cells were fixed and stained as described previously [18]. At least 4 random independent fields in different locations or cells were acquired for each sample. For the migration assays, we used similar inserts without the Matrigel coating. A wound-healing assay was also performed to investigate the migration of the cells. A wound was made in the middle of cells (at 90% confluency) and migration of the glioma was recorded at 24 h.

### 4.8. 3D Culture

Infiltration of cancer cells was also investigated in 3D spheroid cultures. Prior to the experiment, collagen type I (2 mg/mL) and matrigel (11%) were placed in individual chamber slides mixed in cell growth medium. Subsequently, GFP-expressing GBM spheroids were seeded on the collagen-based matrix and covered with the same collagen-based matrix used to make the basement matrix. Infiltration of GBM cells was observed using fluorescence microscopy after 48 h.

### 4.9. qPCR and Western Blot

Protein and RNA from treated samples were extracted using cell lysis buffer and Trizol reagent (Invitrogen) respectively by same protocol as described in our previous report [19] and described in more detail in supplemental procedures. All primers used in this study are listed in Table S1.

### 4.10. Animal Experiments

Glioma cells (1 × 10^6^ cells suspended in 200 μL PBS) were injected into the right hind flank of athymic BALB/c female nude mice (5–6weeks of age; orient) subcutaneously. When the tumor size reaches to 100 mm^3^, mice were randomly divided into plasma treatment and control vehicle groups. For this experiment, plasma activated media (PAM) was prepared with 10 min treatment to incomplete DMEM media by DBD plasma. Plasma treated group of mice received 200 μL of PAM at every other day four times by subcutaneous injection in tumor and the control vehicle group received the same volume of non-plasma exposed medium in similar way. Tumor size was measured with a caliper (calculated volume = shortest diameter^2^ × longest diameter/2) with 3 day intervals. After sacrifice, tumor weight was also recorded in both groups. This study was reviewed and approved by the Institutional Animal Care and Use Committee (IACUC) of the Center for Laboratory Animal Sciences, the Medical Research Coordinating Center and the HYU Industry-University Cooperation Foundation (HY-IACUC-16-0089).

### 4.11. Immunohistochemistry

After sacrifice, control and plasma tumor sample tissues were fixed in formalin for the preparation of paraffin sections. Paraffin-embedded tissue sections were deparaffinized in xylene, 95, 90 and 70% ethanol, followed by PBS. Tissue sections were immunostained overnight at 4 °C with the E-cadherin antibody (1:400; abcam, Cambridge, UK), N-cadherin (1:400; abcam) antibody. After PBS washing, 1:200 dilution of biotinylated goat anti-mouse IgG antibody in blocking solution was applied to the sections and incubated for 30–40 min. Following with PBS, ABC reagent was applied to the sections and incubated further for 30 min. Color reaction was performed with 3, 30-diaminobenzidine (vector laboratories, Seoul, Korea) and slides were washed twice with PBS. After counter-staining with hematoxylin and clearing with graded ethanol series and xylene, sections were mounted with Canada balsam. Images were photographed using IX71 microscope (Olympus, Tokyo, Japan) equipped with DP71 digital imaging system (Olympus). For immunohistochemistry, E-cadherin (ab15148), N-cadherin (ab18203), Vimentin (ab137321), CD86 antibody (NBP2-25208-novus biologicals, Centennial, CO, USA) were used.

### 4.12. Kaplan-Meier Analysis

Patient Survival data were obtained from an online TCGA database based upon low and high expression of TNF-alpha expression (http://www.betastasis.com/glioma/tcga_gbm/).

### 4.13. Statistical Analysis

All results are expressed as the means ± SD of triplicates. Significant differences between groups were analyzed using Student’s *t*-tests. Levels of significance are indicated as * *p* < 0.05; ** *p* < 0.01; and *** *p* < 0.001. Non-significant groups were designated as ns.

## 5. Conclusions

In this report, we demonstrate that cold plasma can function as an immune modulator for immune cell activation that can stimulate M1/M2 macrophage polarization. Particularly, the numbers of mitochondria and lysosomes were increased during monocyte cell differentiation after plasma stimulation and cell surface marker CD86 expression was elevated. Simultaneously, the induction of both macrophage subsets demonstrated the ability of plasma to induce polarization of macrophages. However, TNF-α and IL-12 were found to increase cell death by activated macrophages, which might be the results in the reduction of cellular ATP in GBM cells. Particularly, these M1-polarized macrophages may delay the EMT process and increase expression of the epithelial marker E-cadherin in glioma cells in vitro as well as in vivo. Plasma-polarized macrophages attenuated cancer stem cell maintenance, suggesting a potential role of plasma in switching macrophage function. Delays in the function of anti-inflammatory M2 and increased function of pro-inflammatory M1 macrophages might be key targets in controlling tumorigenesis caused by compromised immune responses.

## Supplementary Materials

The following are available online at https://www.mdpi.com/2072-6694/11/6/842/s1, Figure S1: Uncropped images of western blot used in Figure 2, Figure S2: M1 and M2 markers were increased in plasma-treated THP1 cells. qPCR analysis for CD80, 68 and CD206 gene expression in control and 3 min plasma-stimulated THP1 cells. Error bars represent the mean ± S.D. of triplicate samples. * *p* < 0.05 and ** *p* < 0.01, Figure S3: TCGA data analysis for NOS expression in normal and brain carcinoma subtypes. Box plot pattern of iNOS levels between classical, mesenchymal, Neural, Proneural subtypes. All brain carcinoma subtypes have been compared to normal tissues. Error bars represent the mean ± S.D. of triplicate samples. *** *p* < 0.001, Figure S4: Uncropped images of western blot used in Figure 3, Figure S5: Number of affected transcripts by plasma exposure in differentiated macrophages. (A) Venn diagram of genes expressed in macrophages reporting the numbers modulated in response to plasma exposure. (B) PCA of the transcriptome during macrophages after plasma treatment, Figure S6: Expression of cytokines in cold plasma-stimulated macrophage during M1 and M2 polarization. (A) Release of IL-10 cytokines was evaluated in cell culture media after 24 h of co-culture using ELISA assay in plasma treated THP-1 cells as per indicated panels. (B) qPCR analysis of iNOS in control and 3-min plasma-treated macrophages. Error bars represent the mean ± S.D. of triplicate samples. * *p* < 0.05, ** *p* < 0.01, Figure S7: Cold plasma-stimulated macrophage suppresses the self-renewal of glioma cells. Limiting dilution assay was performed in glioma sphere cells which was co-cultures with plasma-stimulated macrophages, Figure S8. The macroscopic observation of (a) control and (b) plasma activated media (PAM) mice bearing subcutaneous tumors on hind flank, Table S1: Primer sequence are listed used in the study.
