# Supplementary material for: Preventing the Solid Cancer Progression via Release of Anticancer-Cytokines in Co-Culture with Cold Plasma-Stimulated Macrophages
Source: Cancers (Basel). 2019 Jun 18;11(6):842. doi: 10.3390/cancers11060842 (PMC6628390; doi:10.3390/cancers11060842)
Supplement: Supplementary file 1 [file cancers-11-00842-s001.pdf]

Supplementary Information

Preventing the solid cancer progression via release of anticancer-cytokines in co-culture with cold plasma-stimulated macrophages

Nagendra Kumar Kaushik, Neha Kaushik, Manish Adhikari, Bhagirath Ghimre, Nguyen Nhat Linh, Yogendra Kumar Mishra, Su-Jae Lee and Eun Ha Choi

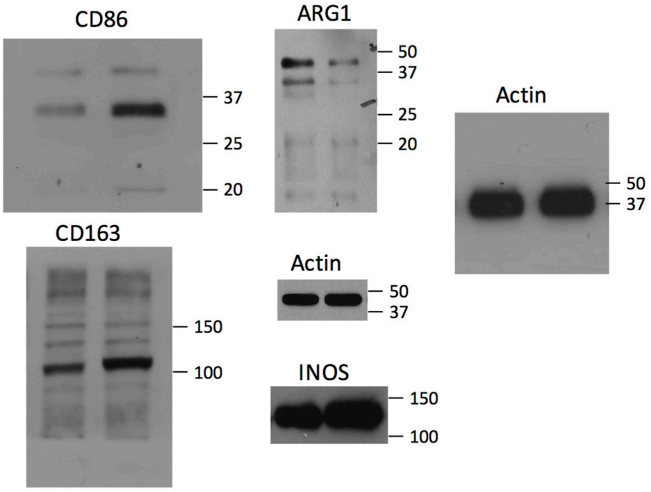

Figure S1. Uncropped images of western blot used in Figure 2.

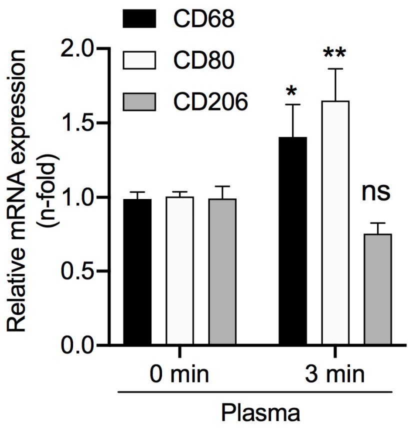

Figure S2. M1 and M2 markers were increased in plasma-treated THP1 cells. qPCR analysis for CD80, 68 and CD206 gene expression in control and 3 min plasma-stimulated THP1 cells. Error bars represent the mean ± S.D. of triplicate samples. * $p < 0.05$ and ** $p < 0.01$.

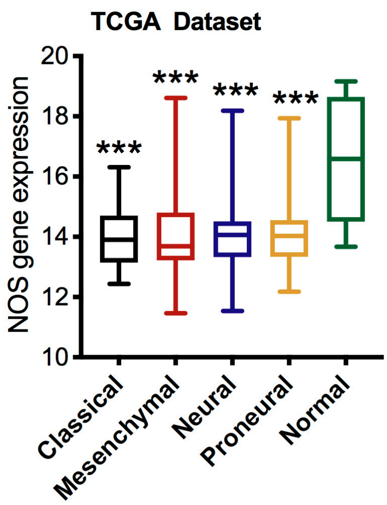

Figure S3. TCGA data analysis for NOS expression in normal and brain carcinoma subtypes. Box plot pattern of iNOS levels between classical, mesenchymal, Neural, Proneural subtypes. All brain carcinoma subtypes have been compare to normal tissues. Error bars represent the mean ± S.D. of triplicate samples. *** $p < 0.001$.

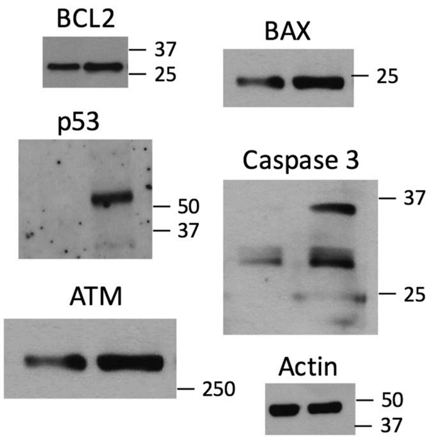

Figure S4. Uncropped images of western blot used in Figure 3.

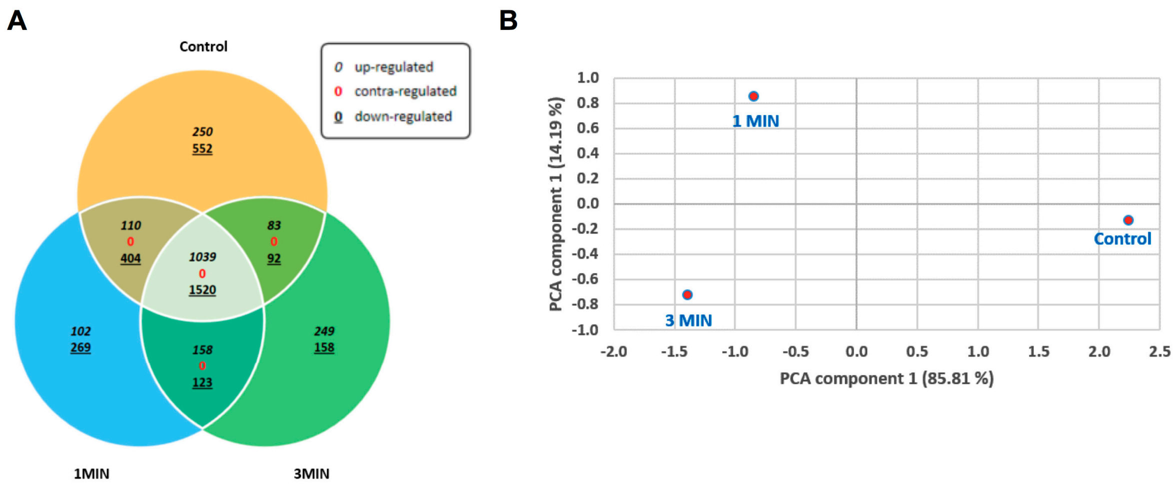

Figure S5. Number of affected transcripts by plasma exposure in differentiated macrophages. (**A**) Venn diagram of genes expressed in macrophages reporting the numbers modulated in response to plasma exposure. (**B**) PCA of the transcriptome during macrophages after plasma treatment.

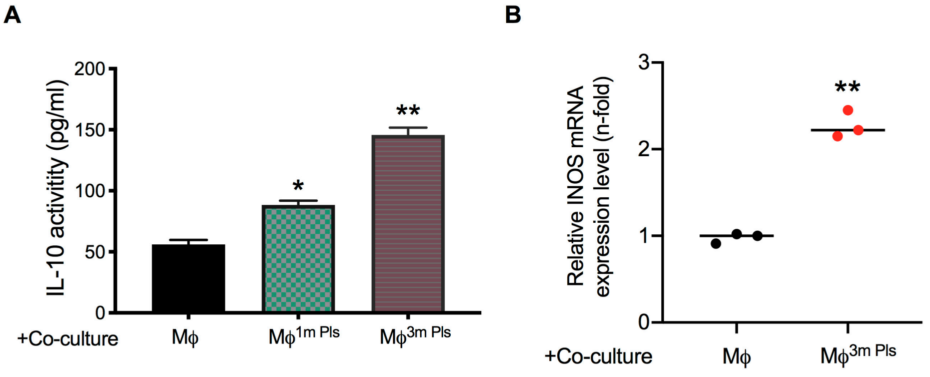

S

Figure S6. Expression of cytokines in cold plasma-stimulated macrophage during M1 and M2 polarization. (**A**) Release of IL-10 cytokines was evaluated in cell culture media after 24 h of co-culture using ELISA assay in plasma treated THP-1 cells as per indicated panels. (**B**) qPCR analysis of iNOS in control and 3-min plasma-treated macrophages. Error bars represent the mean ± S.D. of triplicate samples. * $p < 0.05$, ** $p < 0.01$.

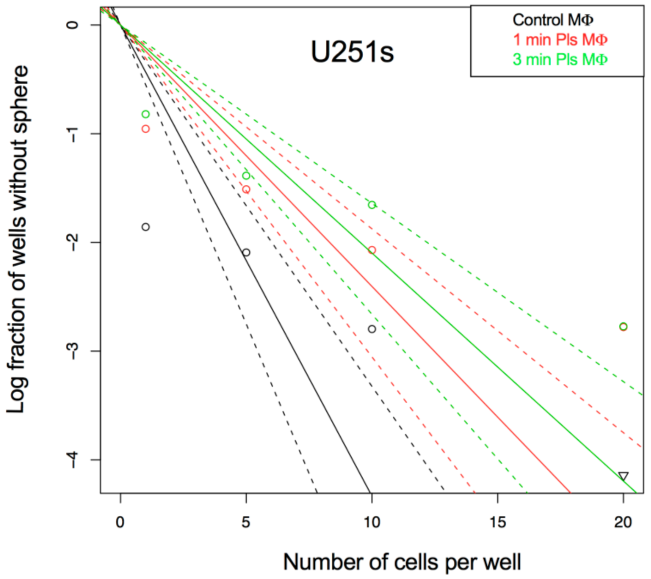

Figure S7. Cold plasma-stimulated macrophage suppresses the self-renewal of glioma cells. Limiting dilution assay was performed in glioma sphere cells which was co-cultures with plasma-stimulated macrophages.

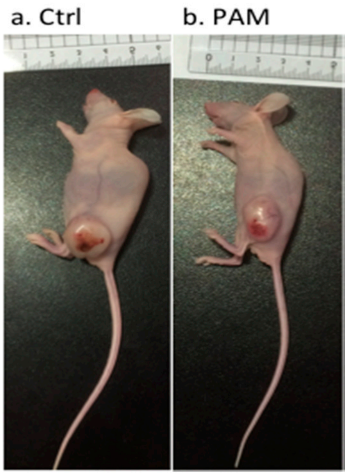

Figure S8. The macroscopic observation of (**a**) control and (**b**) plasma activated media (PAM) mice bearing subcutaneous tumors on hind flank.

Table S1. Primer sequence are listed used in the study.

Gene Name	Symbol	Primer Sequences (5′-3′)	Amplicon Size (bp)
Cluster of Differentiation 86	CD86	L-AGATGTCCTACGGGAACGTG<br>R-ATCCCACCTTAGAGCCAGGT	152 bp
Cluster of Differentiation 163	CD163	L-TGTGGCCTGCATAGAGAGTG<br>R-TTCCCCAAAATGAGCAGAAC	199 bp
Cluster of Differentiation 80	CD80	L- CGAGTACAAGAACCGGACCA<br>R-T TAACGTCACTTCAGCCAGGT	154 bp
Cluster of Differentiation 68	CD68	L- TAGCTGGACTTTGGGTGAGG<br>R- AACTGTGACGTTTCCATGGC	222 bp
Cluster of Differentiation 206	CD206	L- GGCCAAGCTTCTCTGGAATG<br>R- GCCACGTCCCTTCAACATTT	154 bp
Inducible nitric oxide synthase	iNOS	L-TGCTGCTGCTGACCATTAAC<br>R-AGACCCCAACACAAACAAGC	224 bp
Arginase 1	ARG1	L-CCCTTTGCTGACATCCCTAA<br>R-ATTGCCAAACTGTGGTCTCC	155 bp
Chemokine (C-C motif) ligand 1	CCL1	L-TGCTCCAATGAGGGCTTAAT<br>R-GTAGTTTCGGGGACAGGTGA	184 bp
Chemokine (C-C motif) ligand 2	CCL2	L-CCCCAGTCACCTGCTGTTAT<br>R-TCCTGAACCCACTTCTGCTT	166 bp
Chemokine (C-C motif) ligand 4	CCL4	L-GAGTTCTGCAGCCTCACCTC<br>R-ACCACAAAGTTGCGAGGAAG	193 bp
C-X-C motif chemokine 5	CXCL5	L-TCTGCAAGTGTTCGCCATAG<br>R-GAAACTTTTCCATGCGTGCT	183 bp
Interleukin-10	IL-10	L-AGCTGTGGCCAGCTTGTTAT<br>R-GTAGAGACGGGGTTTCACCA	164 bp
Interleukin-1 alpha	IL-1α	L-ATCAGTACCTCACGGCTGCT<br>R-TGGGTATCTCAGGCATCTCC	189 bp
C-C chemokine receptor type 2	CCR2	L-GTGTGTGGAGGTCCAGGAGT<br>R-AAGCCAGACGTGTGATTTCC	181 bp
Chemokine (C-X-C motif) ligand 8	CXCL8	L-AGACAGCAGAGCACACAAGC<br>R-ACTCCTTGGCAAAACTGCAC	162 bp
Chemokine (C-X-C motif) ligand 16	CXCL16	L-CTCCTGGCCATCATCTTCAT<br>R-GAGTCCGTCTCCTCACAAGC	168 bp
Interleukin-1 beta	IL-1ß	L-CGATGCACCTGTACGATCAC<br>R-TCTTTCAACACGCAGGACAG	228 bp
Interleukin-6	IL-6	L-AGGAGACTTGCCTGGTGAAA<br>R-CAGGGGTGGTTATTGCATCT	180 bp
Tumor necrosis factor alpha	TNF-α	L-CCCTGAAAACAACCCTCAGA<br>R-AAGAGGCTGAGGAACAAGCA	217 bp
BCL-2- associated X protein	BAX	L-AAGGCTGAGACGGGGTTATC<br>R-AAGTGCAAAAGCTCAGAGGC	168 bp
Tumor protein p53	P53	L-GGAAATCTCACCCCATCCCA<br>R-CAGTAAGCCAAGATCACGCC	190 bp
Caspase 3	CAS3	L-GCTGGACTGCGGTATTGAGA<br>R-CCATGACCCGTCCCTTGA	142 bp

Experimental Procedures

Antibodies

The antibodies for β-actin (A5316) were obtained from Sigma-aldrich. The anti-CD86 (ab53004), anti-CD163 (ab182422), anti-E-cadherin (ab1416), anti-Vimentin (ab8978), anti-N-cadherin (ab18203), and anti-BAX (ab77566) was obtained from Abcam, and the anti-cleaved caspase-3 (sc-98785), anti-Bcl2 (sc-509), anti-ATM (sc-135663), anti-p53 (sc-47698) antibody was from Santa cruz Technologies

and ARG1 (#9819), iNOS (#13120) was purchased from cell signaling. Western blots were developed using ECL procedures (Amersham, IL, USA).

Cold μ-DBD Plasma Source and Treatment Conditions

The μ-DBD plasma source used to monocyte derived macrophage activation experiment is shown in Figure 1A. It consists of coplanar silver electrodes fabricated on a glass substrate (SiO_2) using photolithography. To prevent the deposition of water molecules above the dielectric surface, a 1 μm-thick hydration prevention layer made from alumina (Al_2O_3) was deposited above the dielectric layer. A detailed description of a similar working device can be found in our previous reports [1]. Figure 1B, 1C and 1D show the optical emission spectrum (OES), live plasma discharge image and current/voltage profile, respectively. Figure 1E shows the structure of the PMA. Monocytic THP1 cells were seeded in 35-mm diameter cell culture dishes containing 200 nM PMA for 48 h, and the medium was replaced with fresh culture medium prior to exposure to μ-DBD surface N_2 plasma discharges (1 min and 3 min) and further incubated for 5 days. A similar dose was applied in all experiments. An approximately 2-mm distance was maintained between the plasma source and the upper surface of the medium in the cell culture dish.

Viability Assay

Cellular viability after co-culture was determined in brain cancer cells using reliable colorimetric MTT assays. All detection steps were performed according to our reported methods [2]. Viable cells were measured after 48 h of co-culture with plasma-treated macrophages.

TUNEL Assays

Glioma cells were grown on coverslips after co-culture with macrophages and stained with APO-BrdU™ TUNEL Assay Kit-with Alexa Fluor®® 488 Anti-BrdU Kit, Molecular probes, Invitrogen. TUNEL staining was performed to label DNA strand breaks for the detection of apoptotic cells. Assay detection steps were performed according to the manufacturer's, protocol.

PARP Assays

Quantitative measurement of the 89 kDa fragment (cleaved fragment) of human PARP-1 was directed in U251MG after co-culture with macrophages cells using an ELISA method provided in the Cleaved PARP-1 In-Cell ELISA Kit (Abcam, Korea).

Protein and RNA Extraction

Total cell RNA was isolated using Trizol reagent (Thermo-scientific). Reverse transcription (RT)-PCR was performed using a Superscript II reverse transcriptase kit (Invitrogen). Every real-time PCR was performed using the CFX96™ Real-Time System in a BioRad machine with the IQ SYBR Green Supermix (BD Biosciences). The primers used in this study were designed and purchased from Macrogen. Primers are listed in Table S1 for detailed information. For western blot analysis, cells were harvested and lysed with RIPA cell lysis buffer (Thermo-scientific) containing protease inhibitors. Briefly, cells were incubated on ice for 30 min and centrifuged at 10000 rpm at 4 °C. Supernatants were collected and immediately used for immunoblotting. Equal amounts of protein of each sample were examined by SDS–PAGE.

Clonal Assays for Stem Cells

The size of spheres was determined using Motic Images Plus 2.0 software in four randomly chosen fields until day 4. In short, individual glioma sphere cells were seeded into 96-well plates in triplicate, and clone formation was recorded for 10 days. Clones were pictured using a phase-contrast microscope, and size was measured by Motic Images Plus 2.0. For the limiting dilution assay (LDA), cells were plated at various dilutions, such as 40 cells/well, 20 cells/well, 10 cells/well, 5 cells/well, 1

cell/well, in 32 wells for each dilution, respectively, in ultra-low-attachment 96-well cell-culture plates. Data were acquired using the online tool available at http://bioinf.wehi.edu.au/software/elda/.

Microarray Data Analysis

In the present study, we performed global gene expression analyses using Affymetrix GeneChip®® Human Gene 2.0 ST oligonucleotide arrays. Total RNA was isolated using Trizol reagent (Invitrogen); the RNA quality was assessed by Agilent 2100 bioanalyzer (Agilent Technologies, USA), and quantity was determined by ND-1000 spectrophotometer (NanoDrop Technologies, USA). Per RNA sample, 300 ng were used as input into the Affymetrix procedure as recommended by protocol (http://www.affymetrix.com). Briefly, 300 ng of total RNA from each sample was converted to double-strand cDNA Using a random hexamer incorporating a T7 promoter, amplified RNA (cRNA) was generated from the double-stranded cDNA template though an IVT (in-vitro transcription) reaction and purified with the Affymetrix sample cleanup module. cDNA was regenerated through a random-primed reverse transcription using a dNTP mix containing dUTP. cDNA was then fragmented by UDG and APE 1 restriction endonucleases and end-labeled by terminal transferase reaction incorporating a biotinylated dideoxynucleotide. Fragmented end-labeled cDNA was hybridized to the GeneChip®® Human Gene 2.0 ST arrays for 16 h at 45 °C and 60 rpm as described in the Gene Chip Whole Transcript (WT) Sense Target Labeling Assay Manual (Affymetrix). For the normalization, an RMA (Robust Multi-Average) algorithm implemented in Affymetrix Expression Console software was used. A principal component analysis (PCA) was performed on the entire set of genes evaluated to assign the general variability in the data to a deceased set of variables called principal components and analyzed using Genowiz4.0TM (OcimumBiosolutions, India) [3]. To categorize overrepresented biological categories within each cluster, the Gene Ontology (GO) database was used for the entire set of genes [4].

References

1. Kaushik, N.K.; Kaushik, N.; Yoo, K.C.; Uddin, N.; Kim, J.S.; Lee, S.J.; Choi, E.H. Low doses of PEG-coated gold nanoparticles sensitize solid tumors to cold plasma by blocking the PI3K/AKT-driven signaling axis to suppress cellular transformation by inhibiting growth and EMT. *Biomaterials* **2016**, *86*, 118–130.
2. Kaushik, N.; Lee, S.J.; Choi, T.G.; Baik, K.Y.; Uhm, H.S.; Kim, C.H.; Kaushik, N.K.; Choi, E.H. Non-thermal plasma with 2-deoxy-D-glucose synergistically induces cell death by targeting glycolysis in blood cancer cells. *Sci. Rep.* **2015**, *5*, 8726, doi:10.1038/srep08726.
3. Raychaudhuri, S.; Stuart, J.M.; Altman, R.B. Principal components analysis to summarize microarray experiments: Application to sporulation time series. *Pac. Symp. Biocomput.* **2000**, *5*, 452–463.
4. Jr Dennis, G.; Sherman, B.T.; Hosack, D.A.; Yang, J.; Gao, W.; Lane, H.C.; Lempicki, R.A. DAVID: Database for Annotation, Visualization, and Integrated Discovery. *Genome Biol.* **2003**, *4*, P3.

© 2019 by the authors. Licensee MDPI, Basel, Switzerland. This article is an open access article distributed under the terms and conditions of the Creative Commons Attribution (CC BY) license (http://creativecommons.org/licenses/by/4.0/).
